# Aerial Flight Paths for Communication

**DOI:** 10.3389/frobt.2021.719154

**Published:** 2021-12-07

**Authors:** Alisha Bevins, Brittany A. Duncan

**Affiliations:** NIMBUS Lab, Department of Computer Science and Engineering, University of Nebraska-Lincoln, Lincoln, NE, United States

**Keywords:** drone, gesture, human-robot interaction, communication, small UAS

## Abstract

This article presents an understanding of naive users’ perception of the communicative nature of unmanned aerial vehicle (UAV) motions refined through an iterative series of studies. This includes both what people believe the UAV is trying to communicate, and how they expect to respond through physical action or emotional response. Previous work in this area prioritized gestures from participants to the vehicle or augmenting the vehicle with additional communication modalities, rather than communicating without clear definitions of the states attempting to be conveyed. In an attempt to elicit more concrete states and better understand specific motion perception, this work includes multiple iterations of state creation, flight path refinement, and label assignment. The lessons learned in this work will be applicable broadly to those interested in defining flight paths, and within the human-robot interaction community as a whole, as it provides a base for those seeking to communicate using non-anthropomorphic robots. We found that the Negative Attitudes towards Robots Scale (NARS) can be an indicator of how a person is likely to react to a UAV, the emotional content they are likely to perceive from a message being conveyed, and it is an indicator for the personality characteristics they are likely to project upon the UAV. We also see that people commonly associate motions from other non-verbal communication situations onto UAVs. Flight specific recommendations are to use a dynamic retreating motion from a person to encourage following, use a perpendicular motion to their field of view for blocking, simple descending motion for landing, and to use either no motion or large altitude changes to encourage watching. Overall, this research explores the communication from the UAV to the bystander through its motion, to see how people respond physically and emotionally.

## 1 Introduction

As UAVs increase in popularity and functionality, they are becoming easier to obtain and significantly more visible in standard occurrences for the general public. In addition to the increase in visibility to the public in everyday occurrences, they are being used in many professional environments such as disaster relief, agriculture, and product delivery. One of the problems with increased visibility and use is that not everyone who comes in contact with the UAV will have context for its purpose or current task. This becomes an even larger issue when a malfunction or abnormality occurs. UAV manufacturers, programmers, and users need to be able to understand how they can expect the uninformed person to react to their vehicle. In addition to this, a bystander needs to be able to understand what is occurring to minimize concern and unnecessary intervention.

**FIGURE 1:**
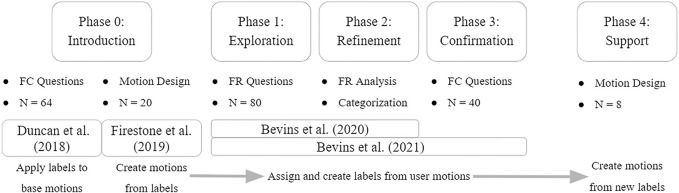
Breakdown of Phases: initial introduction, an iterative creation/labeling exploration, and finally a supporting in-person study.

The main purpose of this work is to inform future researchers, and UAV developers, about how participants perceive UAV paths. This includes what they believe the system to be communicating, the most important components of the flight paths, and their intended reactions based on those communications. To address these issues, we first explore how consistently people label motions (Phase 0). Those labels were then presented to participants to create their own motions to see if there were inherent similarities in these motions (Phase 0). A combined set of motions were then presented to new participants to see if user generated paths had increased label agreement (Phase 1–3). Finally, states which were more effective at generating responses were presented to a final set of participants to understand whether their created motions would align with the expected path characteristics from earlier phases (Phase 4). Figure 1 further introduces the phases and how they will be presented in this paper, in addition to showing prior contributing works.

Overall lessons from this work indicate that:

• frequent motions or gestures applied in non-UAV situations are associated and understood on UAVs,
• landing is conveyed by direct movements with an altitude change,
• people will follow a UAV’s path when the motion approaches and then retreats towards a location when in the absence of altitude changes, and
• flights across an area are likely to cause participants to avoid the vehicle or that area (regardless of the altitude).

We found that simpler motions are more likely to have consistent interpretation across participants. Considering the most basic flight paths, people took the front-back motion on the *y*-axis to mean to follow the vehicle, a side to side motion focused on the *x*-axis to stay back (or to not follow it), and an up-down motion on the *z*-axis to mean landing. We also found that NARS can be an indicator of how people expect to react, if they are likely to expect a negative message to be conveyed, and their expectation for the UAV to have negative personality traits.

## 2 Literature Review

When considering the topics discussed in this paper, the related work is broad and inherits best practices across many fields. This chapter discusses the most relevant work when developing the studies and provides context to those hoping to adopt these practices in the future.

### 2.1 Social UAVs

The work of social UAVs, which we will define as “UAVs that will operate in spaces used by and necessitate communication with human bystanders,” has been expanding rapidly in recent years. This has lead to (Funk, 2018) providing a comprehensive overview of UAVs as flying interfaces, and (Baytas et al., 2019) providing design recommendations for UAVs in inhabited environments. A significant finding from (Baytas et al., 2019) discusses the idea of providing future work on “Intuitive Comprehension” of UAV movements, which means understanding what a UAV is trying to convey without additional explanation. A more comprehensive discussion of social uses for UAV systems can be found in these works.

### 2.2 UAV Communication

(Cauchard et al., 2015; Obaid et al., 2016) have examined different methods to facilitate communication from the human to the UAV. In the work presented here we are more interested in what a UAV can communicate to a person who may or may not be its operator. This can be achieved through a variety of methods, with the most popular discussed further here.

#### 2.2.1 Video, Lights, and Stereo

Audio or video methods can be very direct in their communication by providing speech, either verbal or written, or figures. Attaching a projector onto a UAV is a common video communication method, as demonstrated by (Matrosov et al., 2016; Nozaki, 2014; Scheible et al., 2013). These projects typically project text or video onto an arbitrary object and can also include an interface to allow user control of the display (Matrosov et al., 2016). Merged these uses by creating interfaces projected onto the ground that allowed interactions using motions of a foot. Another visual modality demonstrated for UAV communications are lights (Szafir et al., 2015). Showed the ability to convey robot flight intentions at a glance, specifically to better express directionality. They found participants were able to better distinguish robot predictability over baseline flight behaviors when given four different signal designs.

On the audio side, providing speech to observers is as straightforward as attaching a speaker (Yamazaki et al., 2019). Demonstrated one strong use case by attaching a speaker and microphone system on a UAV that would make sounds for natural disaster victims to react to, and then capture their vocal reactions.

Although most of these studies were more qualitative in nature and had limited participants included, they do show the capability of direct communication from a UAV to a bystander. Unfortunately, adding components to a system always comes with the natural drawbacks of impacting system weight limits and battery usage, which can then in turn impact the system performance. The other drawback for these components is simply that they require additional hardware that is, not standard with most UAV systems. Finally, the methods mentioned here can have a reduced communication range, as they can only communicate as far as their screens can be seen or their speakers heard clearly. Eventually, communications will likely incorporate some of these methods while also leveraging the motion of a UAV, which we will investigate throughout this work.

#### 2.2.2 UAV Proxemics

Proxemics is “the study of how man unconsciously structures microspace—the distance between men in the conduct of daily transactions” as described by (Hall, 1963). It is another component that can be manipulated to assist or change the overall message attempting to be conveyed through a system (Baytas et al., 2019). Discusses the concept of understanding how distancing impacts interaction from a comprehensive view of social UAVs. Other works that have explored the impact of UAV distancing in interactions includes (Duncan and Murphy, 2013; Wojciechowska et al., 2019), who explored using vehicles at different heights (Acharya et al., 2017). Explored that effect using an untethered system in addition to comparing it a ground vehicle. The overall consensus across studies was that interactions within the social zone were preferred to the personal zone, which is in contrast to research with human-human or human-ground robot interactions.

#### 2.2.3 Flight Paths

The benefit of using flight paths for communication from UAV to human has been briefly explored (Sharma et al., 2013). Explored using UAV paths to communicate affective information, and suggested direct vs. indirect use of space and changing the speed of the system are two components that have a direct effect on the valence. From their study they found that a direct quick motion gave higher valence (Szafir et al., 2014). Used flight to assist in communicating intended destination while the system also completed other goals. Overall, they found easing into the motion and arcing it made participants feel the motions were more natural and safe, which is also consistent with the idea that direct, quick motions increased participant valence in (Sharma et al., 2013).

### 2.3 Personality Model

To obtain a richer understanding of how people would respond to a UAV, it is also important to consider their projected emotion in relation to the UAV (Fong et al., 2003). Suggests that stereotype personalities can be created using immediate response emotions (Cauchard et al., 2016; Spadafora et al., 2016) explored this concept and presented an emotional model space for UAVs. Cauchard then also used these models to represent a full personality, or emotional state, such as Brave or Grumpy. These personalities, along with all individualized characteristics, could then be mapped based on varying speed, reaction time, altitude, and additional movement characteristics. Ultimately providing four different stereotypes of personality models that create the Emotional Model Space for UAVs. A few examples of these models include an Adventurer Hero or Anti-Social Drone. Understanding these categories allows us to better match a UAVs’ action to expected action or scenario, in addition to some insight in how they may be perceived.

### 2.4 Affect, Attitude, and Perception

Interactions are biased by our previous experiences and interactions, but it can be difficult to know a participant’s current affective state (and its impact on their study responses) without including a validated instrument. To understand the impact of a participant’s previous experiences on their current interaction, questionnaires can provide this insight. One such instrument to better understand a participant’s affective state and how it changes throughout the study is the Positive and Negative Affect Scale (PANAS) from (Watson et al., 1988). This questionnaire provides insight into how a participant is feeling that day compared to their normal state over the past week, and can be administered post-interaction to examine how the interaction impacts their state. Previous work by (Acharya et al., 2017) suggested that participants may have a higher negative affect after interacting with a UAV. The discomfort with the UAV was also supported by an increased distance in interaction when compared to a ground robot which did not result in an increased negative affect.

Another instrument, the Negative Attitude towards Robots Scale (NARS), has been suggested by (Riek et al., 2010) to impact a participant’s ability to recognize humanoid motions, where participants with more negative attitudes were less able to recognize robot motions. NARS was introduced by (Nomura et al., 2004) and refined by (Syrdal et al., 2009). A participant’s NARS score is calculated by averaging their values for three subcategories: Social/Future Implications, Emotional Attitudes, and Actual Interactions.

### 2.5 Crowdsourcing

Although running in-person studies may typically be preferred, online crowdsouring can be very useful in certain cases. There are a few cases where it may be more appropriate to use a crowdsourcing method. A few examples of these may include: when a large range of participants are needed, materials are targeted for refinement through many different proto-studies, or the work can be delegated into small tasks. Previous work by (Toris et al., 2014; Casler et al., 2013) have compared crowdsourced results to in-person and saw minimal to no difference in their results between the participants who came in person and those who completed tasks online.

## 3 Experimental Methods and Design

This section describes experimental methods, materials, and design which are consistent across the phases of the studies to improve the readability of the article.

### 3.1 Pre and Post Interaction Surveys

Following a consent form, participants completed a demographic questionnaire, the first half of PANAS (based on their test condition, as listed in Table 3), and NARS. After the main task, they all completed a post-survey questionnaire consisting of questions about the study. If they completed PANAS prior to their task, they were asked to complete the second half of PANAS at this time.

### 3.2 Materials

For both Phase 0 studies an Ascending Technologies (AscTec) Hummingbird and Vicon motion capture system were used. For Phase 1–4 we used the DJI flamewheel F450, Pixhawk flight controller, and Vicon motion capture system.

### 3.3 MTurk

It is important to note the constraints on participants who were included in studies that were completed on Amazon Mechanical Turk (MTurk), which includes Phase 0 (Duncan et al., 2018), Phase 1, and Phase 3. Each participant’s condition was dependent upon which of the mTurk task postings they selected. All tasks appeared the same to participants, so they had no insight into any differences and participants were excluded from future tasks once they participated in one. All participants were considered an MTurk “master,” as determined by Amazon through analyzing worker performance over time. Also due to IRB restrictions from the GDPR privacy directive, none of the participants were allowed to be from the European Union.

Following any pre-interaction surveys, participants were redirected to a Google Form where they were asked to watch unique videos of a UAV flying in specific motions. The motions used for each phase are mentioned in their respective sections. Each video was 30 s in length, with repetitions added to reach the desired length if necessary. We used the Exhausted Drone template speed from (Cauchard et al., 2016) and the Anti-Social Drone altitude template to better compare to previous work. During a study participants would randomly be shown an attention check video that had a word displayed in the middle of it rather than simply showing a repeating motion. This check was placed to ensure participants were attending to the questions and watching the majority of the videos.

### 3.4 Motion Design

For the remaining participants, those in Phase 0 (Firestone et al., 2019) and Phase 4, they were presented with proposed states and asked to create motions to communicate those to others. In the case of Phase 0 (Firestone et al., 2019) this study was completed entirely in person. For Phase 4, the design and pre-interaction surveys were administered over Zoom and Google Forms, respectively. Following this they were asked to verbally describe and physically demonstrate their created motions using a small object (either a model drone in Phase 0 or an object roughly the size of a cell phone in Phase 4). The final component of the motion design study in either phase was to observe their drone flights in a Vicon motion capture space before completing the post-interaction survey.

## 4 Phase 0

We now present the initial phase of the project, which includes two different studies. The first study explores label assignment at a high-level, looking for general agreement amongst participants. The second study explores user-defined flights created to convey the labels presented in the first study *via* an in-person setting.

### 4.1 Broad Agreement

Phase 0 (Duncan et al., 2018) involved 64 participants in total (43 Male, 21 Female). 56 identified as Americans, 2 as Chinese, 1 Korean, 1 Japanese, 1 Indian, 2 as “Other,” and 1 did not respond. Each participant was paid 2 dollars and Amazon was paid 50 cents for recruitment. In the two alternative forced-choice (2AFC) task participants were given two labels, one of which was the expected label, and the other was a distractor chosen from a set of seven choices. In the seven alternative forced-choice (7AFC) task they were given all 7 of the options. Participants took 24.63 min (SD = 12.18) in the 2AFC task, and 26.15 min (SD = 12.29) in the 7AFC task.

The goal of this study was to understand if novice users showed broad agreement on the meaning of UAV gestures. To begin we looked to previously established protocols used for human gestures in (Krauss et al., 1991). Krauss looked to understand the level of participants’ agreement by showing them a limited gesture set, followed by a request for them to apply a label from a limited set. Implementing this into a UAV gesture set began by exploring flight paths used by birds in nature and other biologically inspired behaviors, such as in (Arkin, 1998; Murphy, 2000).

#### 4.1.1 Flight Path Labels

Labels were chosen based on flights that generally would require redirection, intervention, or awareness from either bystanders or operators. They were also chosen with the expectation that they would be well understood by novices due to their frequently observed use in other aircraft, being in general common system tasks, and similarity to other states in common technology (such as phones). The final consideration was choosing states that were domain independent, instead of focusing on applications (such as photography). Ultimately, the states chosen were: lost signal, lost sensor, draw attention, landing, missed goal, change position, and low battery.

#### 4.1.2 Flight Path Selection

The original flight path selection was chosen to include motions that had steady periodic motion which could be created from sinusoid functions, to offer the ability to scale, and loop as needed. This in addition to drawing similarities to the biologically inspired avian flight paths originally identified by (Davis, 2000), lead to the eight cyclic motions of: Circle, Figure-8, Left-Right, Loop, Spiral, Swoop, Undulate, and Up-Down. Further details related to these choices and this work in general can be seen in (Duncan et al., 2018).

#### 4.1.3 Results

The results in these studies were judged using a binomial test for 2AFC (compared to 50%) and a chi-squared test (compared to an even distribution) with *p* < 0.01; the resultant necessary agreement was 75% agreement in 2AFC and 34.4% agreement in 7AFC. In the 2AFC test the motions labeled with high agreement included Spiral (Landing, 87.5%), Figure 8 (Lost Sensor, 84.38%), and Swoop (Draw Attention, 75%). In the 7AFC test, 5 motions (3 unique from the first set) were significant at *p* < 0.01. Significant motions were: Circle (Draw Attention, 40.6%), Figure-8 (Change Position, 40.6%), Loop (Landing, 34.4%), Spiral (Landing, 59.4%), and Undulate (Draw Attention, 34.4%).

The full chi-squared values for the 7AFC are *χ* ^2^(6, *N* = 32) = 23, *p* < 0.001 for Circle, *χ* ^2^(6, *N* = 32) = 22.6, *p* < 0.001 for Figure 8, *χ* ^2^(6, *N* = 32) = 12.6, *p* = 0.049 for Left-Right, *χ* ^2^(6, *N* = 32) = 19.4, *p* = 0.003 for Loop, *χ* ^2^(6, *N* = 32) = 50.6, *p* < 0.001 for Spiral, *χ* ^2^(6, *N* = 32) = 11.8, *p* = 0.066 for Swoop, *χ* ^2^(6, *N* = 32) = 15.8, *p* = 0.01 for Undulate, and *χ* ^2^(6, *N* = 32) = 9.4, *p* = 0.15 for Up-Down.

Due to the number of chi-squared tests conducted, we are using the Bonferroni Correction to address possible effects found due to chance. Using this correction, our *p*-values will need to be below 0.0014 to still be considered significant at the same significance level, rather than below 0.01. Using this adjustment, only Circle, Figure 8, and Spiral are still considered significant.

Results overall showed a stronger understanding for Landing to be communicated by a spiraling path, and in general participants gravitated towards states that were less technical. This is shown from having stronger agreement for Draw Attention and Landing, and lower agreement for Lost Sensor. This finding shows support for the need of better refined labels that are commonly understood. Also due to the overall lack of strong agreement, this work suggested exploring open-ended responses and user generated flight paths. Finally, after initial observations, further research is needed to see if a negative NARS score suggests a decreased understanding of a UAV’s motion, similar to the finding for humanoid robots in (Riek et al., 2010), or if a version of NARS should be revised to apply specifically to UAVs. The flight paths, the labels, and the application of NARS are investigated in the remainder of the paper.

### 4.2 Motion Elicitation

Phase 0 (Firestone et al., 2019) presented the same states from the earlier Phase 0 (Duncan et al., 2018) to twenty in-person participants (10 Male, 10 Female) who were local to the testing location in the United States. The cultural breakdown included 10 Americans, 2 Korean, 2 Indian, and 1 of each of Hispanic, Mexican, Austrian-American, Russian, European, and “other.” As an incentive for participation they were each put into a drawing for a chance to win a $25 gift card. The seven states provided to participants were: Attract Attention, Sensor Lost, Low Battery, Signal Lost, Area of Interest, Missed Goal/Target, and Landing.

After eliciting a total of 140 gestures, a taxonomy was created to group the motions according to specific, common characteristics. This taxonomy encapsulates many different categorization/classification techniques. One of the most popular being the Laban Effort System best represented here by the complexity and space categories, from (Ruiz et al., 2011; Chi et al., 2000) respectively. Sharma et al. (2013) also previously used these two characteristics of the Laban Effort System to explore how they impacted people’s perception of robotic motions, specifically flight paths. These categories are also well reflected within categories mentioned throughout (Venture and Kulić, 2019). This taxonomy is presented in Table 1.

**TABLE 1:** Taxonomy for UAV flight classification.

Taxonomy for user-designed flight paths		
Complexity	Simple	Single movement
	Compound	Collection of movements
Space	Direct	Focused approach to a point
	Indirect	Deviates from direct path
Cyclicity	Cyclic	Repeated motion (same path)
	Random	Singular flight path
Command	Roll	Left or right movement
	Pitch	Forward or back movement
	Yaw	Rotation
	Throttle	Up or down movement
Altitude	Increasing	Increase flight height
	Decreasing	Decrease flight height
	Variable	Increase and decrease
	Stable	No height change
Motion	Rectilinear	Only straight movement(s) and 90-degree turns
	Curvilinear	Only curved movement(s)
	Rotational	Only rotates
	Combinational	Combination of the above

The designed gestures were also grouped with common features according to the taxonomy, in addition to common motion characteristics. The most significant groupings were from Landing (thirteen people assign it as descending), Area of Interest and Missed Goal/Target (both had horizontal circles), and Low Battery (up-down motions).

A primary limitation of this work was the relative simplicity in a majority of the designed flight paths. This limitation was addressed in Phases 1–4 which followed to understand whether the difficulty in path creation was due to limited understanding of possible flight paths, difficulty with the initially defined states, or other limitations imposed by the experimental design.

## 5 Exploration: Phase 1

Based on the findings from the Phase 0 explorations into how participants would use a drone’s motion to communicate, we embarked on an iterative approach in hopes of refining and collecting the different possible responses to drone motions. Further detail can be found in (Bevins and Duncan, 2021). A subset of motions demonstrating agreement from both studies in Phase 0 were presented to participants who were asked to respond to different questions about what they believed the drone was communicating and how they may respond.

### 5.1 Approach

The goals of this work were to validate the proposed videos for participant agreement, prototype questions for ability to elicit consistent responses, and understand the impact of asking multiple questions on participant responses. Throughout the study, other interesting considerations were encountered including the impact of pre- and post-questionnaires on the quality of participant responses, which is not central to the understanding of the motions, but is described further detail in (Bevins et al., 2020). The questions and processes developed were then used to better understand participants’ expected perception and anticipated reaction to UAV flight paths. The full list of questions are presented in Table 2, with “Question Type” referring to the participant’s anticipated response type. All of the questions were looking to obtain realistic answers to how participants’ expect to perceive and/or react to a UAV’s motion.

**TABLE 2:** Study questions, with their anticipated response type, assigned number, and character length.

Question number	Question type	Question(s)	Characters
1	Speech	If you saw this drone in real life, what would it say to you?	61
2	Speech	If this drone could speak what would it tell you to do?	55
3	Gesture	What human gesture does this remind you of?	43
4	Gesture	If you had to replicate this movement with your head and/or body, what would you do?	84
5	Physical	If you were in the room with the robot, what would you do immediately following the robot’s action?	99
6	Physical	If you were in the room with the robot, how would you respond immediately following the robot’s action?	103

#### 5.1.1 Question Variants

Three question types, each with two variations, were used in an attempt to obtain convergent responses with respect to participants’ expected reactions. Gesture questions were expected to elicit participants’ relation of the motion of the UAV to an action they have previously observed. Speech questions sought an anticipated verbal communication assigned to the UAV’s motions. Physical questions sought to capture both speech and gesture aspects of the motion, in addition to a possible physical response. For this phase, participants would answer either 1 or 2 questions in a free-response method. The questions chosen are shown in Table 2. The full list of test conditions used, which question(s) were included in each condition, and whether that condition administered PANAS is shown in Table 3. Each line represents 8 participants.

**TABLE 3:** Question combinations for all test conditions within Phase 1.

Test condition	Question numbers asked	PANAS used
1 Speech	1	Yes
1 Speech	1	No
2 Speech	1, 2	Yes
1 Gesture	4	Yes
1 Gesture	4	No
2 Gesture	3, 4	Yes
1 Speech, 1 Gesture	1, 4	Yes
1 Speech, 1 Gesture	1, 4	No
1 Physical	5	Yes
1 Physical	6	Yes

### 5.2 Participants

Phase 1 had 80 participants in total (46 Male, 33 Female, 1 No Answer), with an age range of 24–68 (M = 38.6, SD = 10.7). Of the 80, 76 identified as American, 3 as Indian, and 1 as Chinese. The education levels were: high school (12), some college without a degree (17), college degree (46), and graduate-level degrees (4). Each participant was paid 4 dollars and Amazon was paid 1 dollar for recruitment. Across all of the conditions, participants took roughly 35 min.

#### 5.2.1 PANAS

When examining the initial data that was collected from MTurk, the participants seemed to produce less diverse results towards the end of tasks (particularly those with single questions and double videos). To investigate the possible impact of participant fatigue, we removed the PANAS and additional videos during retests of selected conditions. All test conditions are listed within Table 3.

### 5.3 Videos

**FIGURE 2:**
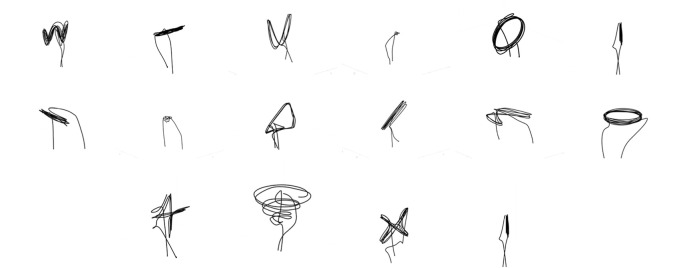
Flight paths from top left to bottom right: undulate, left-right, U-shape, hover, vertical circle, up-down/descend, front-back, yaw, descend and shift, diagonal descend, horizontal figure 8, horizontal circle, plus, spiral, X-Shape, and up-down.

Participants were asked to watch 16 unique videos of a UAV flying in specific motions chosen and created from Phase 0 (Duncan et al., 2018) and Phase 0 (Firestone et al., 2019). This included all of the motions from Phase 0 (Duncan et al., 2018), complemented with a set of motions demonstrating the taxonomic differences and most popular flight paths from Phase 0 (Firestone et al., 2019). The base flight paths included: front-back, straight descend, descend and shift (descend then shift horizontally), diagonal descend, horizontal figure 8, horizontal circle, hover in place, left-right, plus sign, spiral, undulate, up-down, U-shape, vertical circle, X-shape, and yaw in place. Visualisations of these flight paths can be seen in Figure 2. Videos were each 30 s long, and if necessary repetitions of the flight were added to reach the desired length of the video. The paths were held constant for speed, around 0.5 m/s, and overall distance covered was held constant as much as possible. Depending on their condition, participants would see each video either once or twice. It was necessary to repeat a video set when they were asked two questions from the same category (two speech or two gesture). With each video they would receive either 1 or 2 questions. Each time they were asked to watch the entire video, but did have the capability of answering the question and proceeding, as there was not an attention check on every page.

### 5.4 Free Response Question Findings

An analysis of the question results sought to understand which question and/or question type produced the most actionable answers. Specifically, “actionable answers” referred to responses that indicated an intention for verbal or physical response to UAVs. The question type which proved most effective towards this goal was the “Physical” type. Since both questions of this type elicited similar results and only one was needed, we proceeded with “If you were in the room with the robot, how would you respond immediately following the robot’s actions?” Further rationale for this decision is provided in (Bevins et al., 2020). For the purpose of the results presented here, and analysis within Phase 2, the responses were collapsed to be viewed as a single set. This choice was made due to the fact that responses in general were consistent enough for initial analysis, and seemingly more related to the flight path rather than the question.

## 6 Refinement: Phase 2

Through the methods described in this section, an analysis of the data from Phase 1 was conducted to determine which labels contained the most information, in addition to which question would be most effective. This section discusses that process and the steps taken for refinement in future phases.

### 6.1 Frequency Analysis

For an initial understanding of the content of the responses, the 80 participants’ responses from Phase 1 were roughly grouped based on the most commonly used words and general intent behind the words. An example of intent-based grouping would be how the words “stand” and “still” would both be sorted into a stare/observe type of category. From these methods we found 13 prominent categories that covered most of the expressed concepts which are listed in Section 6.2. Through this method we also found that many of the responses had participants describing the motion in some way, such as with “back” for front-back (25), “around” for yaw (20), and “side” for left-right (17). In addition to this, it was common to associate a motion with a human gesture that already exits, such as “nodding” for up-down (12) and “cross” for plus (6).

### 6.2 Category Formation

In addition to the states defined in the Frequency Analysis section, we incorporated categories that represented states such as delivery which are expected to be conveyed within UAV research. In most categories, multiple similar actions were combined to give raters a better sense of the types of responses that could be reasonably grouped together. The full list included:

• Follow/Follow a Path
• Blocked/Stop/Restricted/Do Not Pass
• Go Away/Back Away/Leave
• Move Towards/Approach
• Yes/Approval/Accept/Nodding
• No/Nagging
• Welcome/Hello
• Land/Falling/Lower
• Delivery
• Help
• Watch it/Caution/Slow Down/Investigate
• Stare/Hover/Look/Observe
• Power off

Two raters were asked to categorize the responses based on the provided categories. The raters were given instructions to choose a category only if they believed it appropriately fit, but to otherwise choose “Other.” The raters ended up with kappa agreement scores over 0.93 for all of the categories, which shows excellent agreement (Landis and Koch, 1977). Overall, the goal for this method was to augment the findings from the frequency analysis to generate potential labels in future phases.

Looking into categories when responses are sorted by video provides a few further insights. 15/80 classified hover as “Stare/Hover/Look/Observe.” 10/80 of front-back, 11/80 of horizontal circle, and 11/80 horizontal figure 8 were all classified as “Go Away/Back Away/Leave.” 11/80 straight descend as “Land/Falling/Lower,” 8/80 undulate responses sorted into “Blocked/Stop/Restricted/Do Not Pass,” and finally 8/80 vertical circle as “Watch it/Caution/Slow Down/Investigate.”

#### 6.2.1 Forced Choice Definition

Following the raters’ categorization, the categories were kept for inclusion if they showed high agreement and participant preference. Every category except for “Power Off” ended up being presented to the participants in Phase 3. From the categorization we also noticed that a second question for the participant would be beneficial to elicit answers in all of the categories, and provide more insight into how participants expected to respond. For this reason Question 1, “If you saw this drone in real life, what would it say to you?” was added to Question 6 when designing for Phase 3. The category options were then split across the two questions in an attempt to obtain convergent ideas between the two of them, while also allowing a comparison of the perceived communication with the intended reaction.

Five of the responses were appropriate choices for how participants plan to physically respond to a UAV: “Watch it/Look at it/Stare,” “Investigate,” “Follow it,” “Move Away,” and “Help it,” in addition to an Other category.

The remaining categories were well suited for a speech category question because they helped communicate the states being conveyed to the person rather than showing a reaction to them. Since the responses being chosen here were states that could be communicated, a few of the categories were placed as response options in similar forms to both questions. All response options for Question 1 are: “To Follow It/Move Towards,” “Do Not Follow/Do Not Pass/Restricted/Go Away,” (DNF) “Yes/Approval,” “No,” “Welcome,” “Landing,” “Delivery,” “Help,” and “Caution” in addition to an added Other category.

## 7 Confirmation: Phase 3

Following the refinement phase, we were able to present a new set of participants with the newly generated labels and questions defined in Phase 2 from the data collected in Phase 1. Phase 3 consisted of 40 participants (19 Male, 20 Female, 1 No Answer), ranging in age from 25 to 57 (M = 39.1, SD = 8.1). Of the 40, 33 identified as American, 2 Chinese, 2 Indian, 1 Mexican, 1 Korean, and 1 did not answer. Each participant was presented with the 16 videos, for which they were asked to answer Questions 1 and 6 using the forced choice responses provided in Section 6.

A chi-squared test compared to an even distribution was used to find the statistically significant responses at *α* = 0.01 with the participants from Phase 3, given a null hypothesis that all of the states should be chosen equally. All responses within Table 4 (excluding yaw and the RFP rows) and in Table 5 (excluding the RFP rows) reports significant results at the original given threshold. Similarly to the data presented in Section 4.1.3, due to the number of chi-squared tests conducted we need to address possible effects found due to chance. One way of addressing this is to use the Bonferroni Correction. Using this correction, our *p*-values will need to be below 0.000625 to still be considered significant, rather than below 0.01. Taking this into consideration, all responses within Table 4 excluding Plus, Yaw, and the RFP rows are considered significant. All responses in Table 5 excluding Undulate, Vertical Circle, and the RFP rows are considered significant. The effect sizes and *p*-values are provided in each section.

**TABLE 4:** Q is Quantity of People providing that response, DoF is degrees of freedom, Sample Size is the total number of participants, and RFP refers to rotated flight paths with results only discussed in Section 10.

Motion	Say: Winning Response(s)	Q	DoF	Sample Size	Chi-Square Statistic	*p*-value	Cramer’s V (effect size)
Undulate	Do not follow/Do not pass/restricted/go away (DNF)	14	9	40	52	*p* < 0.0001	0.360
Left-Right		14			50	*p* < 0.0001	
Horizontal Figure 8		14			54.5	*p* < 0.0001	
Horizontal Circle		15			42.5	*p* < 0.0001	
X-Shape		15			41	*p* < 0.0001	
U-Shape		13			33.5	*p* = 0.0001	
Hover		12			29.5	*p* = 0.0005	
Plus		11			23.5	*p* = 0.0052	
Vertical Circle		13			33	*p* < 0.0001	
Up-Down	Yes/Approval	15			39.5	*p* < 0.0001	
Spiral	Tie: DNF	10			37	*p* < 0.0001	
	Tie: Landing						
Front-Back	To Follow It/Move Towards	23			124.5	*p* < 0.0001	
Yaw	Caution	7		32	13.5	*p* = 0.1412	0.528
Descend and Shift	Landing	21			92.5	*p* < 0.0001	
Diagonal Descend		23			112.5	*p* < 0.0001	
Straight Descend		22			103	*p* < 0.0001	
RFP: Undulate	DNF	5		8	22	*p* = 0.0088	0.429
RFP: Rotated Figure 8		4			16	*p* = 0.0669	
RFP: X-Shape	DNF/Landing	2			6	*p* = 0.7399	
RFP: U-Shape	DNF/Landing/Help	2			8	*p* = 0.5341	

**TABLE 5:** Q is Quantity of People providing that response, DoF is degrees of freedom, Sample Size is the total number of participants, and RFP refers to rotated flight paths with results only discussed in Section 10.

Motion	Respond: Winning Response(s)	Q	DoF	Sample Size	Chi-Square Statistic	*p*-value	Cramer’s V (effect size)
Undulate	Move Away	15	5	40	20.86	*p* = 0.0008	0.375
Left-Right		17			29.43	*p* < 0.0001	
Horizontal Figure 8		15			22.57	*p* = 0.0004	
Horizontal Circle		18			30.29	*p* < 0.0001	
X-Shape		18			32.00	*p* < 0.0001	
U-Shape		17			32.57	*p* < 0.0001	
Spiral		19			39.14	*p* < 0.0001	
Plus	Watch it/Look at it/Stare	15			29.71	*p* < 0.0001	
Vertical Circle		14			18.85	*p* = 0.0020	
Up-Down		16			25.43	*p* = 0.0001	
Hover	Tie: Watch it/Look at it/Stare	14			30.29	*p* < 0.0001	
	Tie: Move Away						
Front-Back	Follow It	15			26.29	*p* < 0.0001	
Yaw	Watch it/Look at it/Stare	13		32	22.33	*p* = 0.0004	0.424
Descend and Shift		15			32.33	*p* < 0.0001	
Diagonal Descend		14			35.66	*p* < 0.0001	
Straight Descend	Move Away	12			25.00	*p* = 0.0001	
RFP: Undulate	Move Away	4		8	16.00	*p* = 0.1562	0.547
RFP: X-Shape		3			8.00	*p* = 0.5494	
RFP: Rotated Figure 8	Tie: Follow it	3			14.00	*p* = 0.2206	
	Tie: Move Away						
RFP: U-Shape	Watch it/Look at it/Stare	3			10.00	*p* = 0.4158	

### 7.1 Perceived Communication

This section further discusses the results from the question “If you saw this drone in real life, what would it say to you?” In general, most participants assigned either DNF or “Landing.”

The results presented in Table 4, suggest that participants would perceive a UAV to be blocking a path given large movements across the *x*-axis, with or without movement in the *z*-axis as well. Simpler motions with altitude changes were strongly associated with the intent to communicate “Landing.” When the motions became more complex, incorporated a second direction (descend and shift), or additional axis of motion (spiral) it was not understood as clearly to mean “Landing” even though the dominant motion was within the *z*-axis.

There were a total of 640 responses, the breakdown of responses is represented by 25.7% responses for DNF, 15.9% for “Landing,” about 13% for both “Caution” and “To Follow It/Move Towards,” and 7.5% or less for each of the remaining categories. These values demonstrate that some categories are more likely to be chosen while others are either not well-defined or not anticipated to be associated with drone motions. These values are presented across all videos, but the distribution by video can be seen in Tables 4, 5.

### 7.2 Anticipated Physical Response

Question 6, “If you were in the room with the robot, how would you respond immediately following the robot’s actions?” the second question asked of participants saw a majority of responses for “Move Away” or “Watch it/Look at it/Stare,” with the only significant deviation being front-back receiving an answer of “Follow it.”

Some significant motion traits that appear when analyzing the responses for this question include “Watch it” responses having a key motion along the *z*-axis or not having movement along any of the axes. Vertical circle, descend and shift, yaw, up-down, plus, and diagonal descend all demonstrate this trend. Most of these motions also have a second highest choice of “Move Away,” which likely explains the dissent within the straight descend and spiral paths. For these two specific motions, the popular choice was more evenly split between “Watch it” and “Move Away,” of which the latter ultimately won out. The main takeaway from these results is that we can assume people would either watch or move away from vehicles that are relatively static or undergoing large altitude changes. “Follow it” was most prominent only with movements that were focused on the *x*-axis or x-y plane and approached closer to the participant, as shown with front-back and horizontal figure 8. This led to an additional exploration of the RFP motions which is presented in Section 10.2.

Of the 640 responses, the breakdown of responses is represented by 36% of the responses were for “Move Away,” 30% “Watch it,” 18.8% “Investigate,” 10.4% “Follow it,” 4.5% “Help It” and Other was only chosen once for hover.

### 7.3 Free Response Within Forced Choice

With each of the questions participants had an “Other” option they could fill in if they felt none of the forced choice responses provided accurately portrayed their intentions. There were 13 total write-ins, accounting for a total of about 2% of the responses. None of the motions received more than 4 write-in answers. 12 in total were written in for the perceived communication question from 8 different people, and only 1 answer was written in for the anticipated physical response question. Responses varied in content, but searching, confusion, and watching were popular among the write-ins.

## 8 Phase 4

As an exploration to support the results from earlier phases, we presented 8 participants (6 Male, 2 Female) with the 8 communicative states used in Phase 3 to observe whether their motions would agree with the findings of Phase 3. Prior to participating these participants agreed to both an online and in-person session, so they are all local to the testing location in the United States.

Participants were asked to create flight paths to communicate states from Phase 3, similar to the methods of (Firestone et al., 2019), but over Zoom instead of in-person. Following this, they were expected to come in-person to view their flight paths on a real UAV, but for various reasons not all were able to complete the viewing portion of the study. This section also discusses the work of Phase 4 as compared to the other phases and related works.

### 8.1 Methods

After being greeted and consented, participants were asked to “please design an appropriate gesture, a flight path, for a drone to fly to communicate the state” for each of the states. After designing an appropriate gesture, they were asked to specify details about their motions, such as specific height, speed, and characteristics they would apply to their motions. They filled out a Google Form to answer all of these questions before verbally describing and physically demonstrating their motion using a small object of their choice (around the size of a cell phone).

#### 8.1.1 Height

Participants were given the options of “Above Head,” “Eye Level,” “Chest Level,” “Waist Level,” “Knee Level,” “Ground,” and “Other” to associate with each motion. Due to low response choices, the options for waist, knee, and ground were grouped together for discussion. Table 6 shows the full breakdown of heights chosen, sorted by state.

**TABLE 6:** Participants’ chosen height of operation by state.

	Above head	Eye	Chest	Waist and below	Other
Do not follow/Go away	1	3	2	1	1
Watch it/Look at it	1	5	1	0	1
Investigate	2	1	4	1	0
Caution	2	1	4	1	0
Follow it/Move towards	2	1	5	0	0
Yes/Approval	3	3	2	0	0
Landing	1	2	1	3	1
Delivery	2	1	0	4	1

#### 8.1.2 Speed

Participants were given the options “Fast,” “Average,” “Slow,” and “Other” as options for their chosen speed. No further details about what concrete speed these choices entailed were provided. All eight participants answered this question for the majority of motions, but one chose “Other” for Do Not Follow, and another did not answer the question for Follow it. Table 7 shows the full breakdown of speeds chosen, sorted by state.

**TABLE 7:** Participants’ chosen speed of interaction by state.

	Fast	Average	Slow
Do Not Follow/Go Away	4	1	2
Watch it/Look at it	0	4	4
Investigate	1	3	4
Caution	1	3	4
Follow It/Move Towards	1	4	2
Yes/Approval	2	5	1
Landing	1	2	5
Delivery	0	4	4

#### 8.1.3 Size and Space of UAV

Since participants created the gestures online they had no concept of where these motions would be used (i.e., indoor/outdoor) and thus how much space their UAV would have to fly. Some people created motions that were either fully or slightly dependent upon the space that the UAV was flying in. For example, one person created motions that should go to the extremity of a person’s view (fly as far as the operator could see it), or to the extremity of an available space (edges of a room). A more frequent response was to slightly scale up motions for a larger space/interaction area or larger UAV. The size of the UAV was also left open-ended, this appeared to cause some participants to think of the UAV as the size of the object they were holding.

#### 8.1.4 Excluded Participants

In the early trials of running the Phase 4 study, and in response to the limitations identified from Phase 0 (Firestone et al., 2019), the experimenter showed brief demonstrations of possible flight characteristics. Due to anomolies in their responses this resulted in two participants, in addition to the eight described earlier, being excluded from the results, and analysis presented here in case they were unknowingly biased by the experimenter. During their task descriptions one participant was shown a circle and the other was shown line movements along axes. Both of these participants then showed these demonstrated characteristics consistently within their created flight paths. For the participant shown the circle 6/8 of their motions were categorized as curvilinear, and for the participant shown axis movement all of their motions were categorized as rectilinear. The remaining participants were not shown any example flight demonstrations. This exclusion raises significant concern on how seemingly small differences in experimental design with nascent technologies can unwittingly prime participant responses.

### 8.2 Results

We had participants recommend their preferred characteristics for an entire interaction space, including speed, height, and motion. The designed interactions section below provides a summary for each of the states, in addition to participants’ speed and height characteristics.

#### 8.2.1 Designed Flight Paths

Starting with “Do Not Follow/Go Away,” five participants created different variations of a motion retreating from them, in addition to that two others chose small back-forth juts. This later motion is well reflected in the dominant speed trait, with fast being the most popular choice. For this motion it was also most common to place it around eye level.

For “Watch it/Look at it,” two participants chose a yaw motion, for this motion we also see the first dynamic designs. With participants creating motions that either circled, created a diagonal line, or yaw towards the object of interest. There were also designs involving all three of those motion components that did not have a mentioned attachment to a specific area or object to observe. Five participants designed motions that they placed at eye level, and split their speed preference evenly between average and slow.

For “Investigate” the most dominant trait having movement along the x-y plane. Four of these motions involving a circle, three of which were horizontal. Most of them contained a line either moving left-right or front-back, but not both. Both Investigate and Caution were placed at a majority of chest level (with some eye level and above), and have a split for speed between average and slow. Looking deeper into the per-person breakdown shows that even though these two ended up with the same distribution, many of the participants chose different answers for each one (i.e., the same people didn’t pick the same answers for both). The motions for “Caution” also don’t have any curvilinear characteristics, and while three people designed a left-right motion, three more people also designed a vertical motion (up-down, vertical triangle) indicating further differences between the two states.

The “Follow it/Move Towards” motions, similar to the “Do Not Follow/Go Away,” had six people create motions that moved away from the person. In these cases though the motions were more dynamic. A great example of this from one person is that they wanted the motion to make a line towards their destination with periodic yaws back towards the person. The remaining two suggested up-down changes. Overall the speed and height also show distinction between the two states. People here wanted the motion to be at chest level rather than eye, and chose an average speed rather than fast. This speed difference could indicate more of an offer for guidance (particularly paired with the yawing to ensure following) rather than fleeing in the earlier state.

For “Yes,” all eight designed motions in the vertical plane, four of which were simply an up-down motion. Participants commonly noted that a reason for this was because it matches current human non-verbal communication in nodding or because it matches yes in sign language. These motions were placed at above head/eye levels with an average speed.

“Landing” also showed high agreement among participants, with six including a down motion, three of which were straight down. All of the motions involved the vertical plane, and two of them incorporated a yaw component. Every height category received placement, with slight majority going to waist and below, but there is much greater agreement that the motion should be slow in speed.

Finally, “Delivery” involved four participants designing an approach and three including a curved motion in various ways (curved approach, vertical circle, and “D” shape). Again placing the height at waist and below, and speeds of average or slow.

A couple of people mentioned when choosing motions placed below eye level that they wanted to be able to clearly see the UAV. One person described having it fly at this lower height gave them what felt like more control over the situation.

### 8.3 In-Person

Five of the eight participants were able to come in-person to view their created gestures performed by a DJI Flamewheel F450, the same vehicle that was used in the video recordings and in the same lab space used throughout the phases. After viewing their motions, they were asked if they would change anything. Most did not request any major changes to their originally designed motion, but all five mentioned changes they would make to at least one of their motions after viewing.

Typically these changes were in relation to the overall size of the motion, such making it larger or smaller. The amount that these motions were made larger was not consistent or a direct multiple of their small demonstration object to the size of the UAV. One participant designed motions that were originally proposed to be six inches in size, after viewing they determined it was not as clear as they desired. Another participant’s motion was originally proposed to be approximately one foot and instead requested a change to six feet. One hypothesis for the large number of requested size changes was because participants may not have considered that a UAV, even in a highly controlled space with a Vicon system, has small perturbations while hovering. Because of this noise, the smaller motions were not usually large enough to create a clear distinction for their specific motion. Besides size changes, the only other change of note was when a participant requested to have the UAV move away from them rather than towards in all motions they designed with an approach (Watch it/Look at it, Investigate, Caution, and Delivery).

#### 8.3.1 Added Modalities

At the end of their interaction each person was asked if they could add any modality to the UAV, what it would be. The responses were: Speaker/Sound x2, LED Panel x2 (green = good, red = bad/stop) (green = follow me, yellow/orange = caution), and an on-board distance sensor to have the ability to act with a perception of the space around them.

### 8.4 Comparison to Previous Work

Phase 4 was run explicitly to compare to previous work in Phases 0–3 and to prior work by other colleagues working in this area. In this section, we will describe where this phase supports or contradicts work that has come before and then present areas that are well motivated for future studies.

#### 8.4.1 Comparison to Phase 0 (Label Creation)

Only two of the states included in this phase were presented in Phase 0 (Duncan et al., 2018), “Landing” and “Draw Attention” which map to “Landing” and “Watch it/Look at it.” The methods for Phase 4 are significantly different than in (Duncan et al., 2018), so any support is likely to be only *via* high-level flight path characteristics. Examining commonalities in responses between these works, we can see that all motions with a draw attention label (Circle, Loop, Swoop) are curvilinear, which we also see = in two of the eight motions designed for the “Watch it/Look at it” state. For “Landing,” while one person did create a spiral in Phase 4 for landing, more common is a significant movement along the *z*-axis. From the similar characteristics found between the two works, we see very light support for Phase 0 (Duncan et al., 2018) results from Phase 4.

#### 8.4.2 Comparison to Phase 0 (Gesture Elicitation)

The motions created by participants in Phase 4 were all categorized according to the taxonomy presented in Phase 0 (Firestone et al., 2019), and shown in Table 8.

**TABLE 8:** Motions created in Phase 4 classified according to the taxonomy and labeling from (Firestone et al., 2019).

State	Complexity	Space	Cyclicity	Command	Altitude	Motion
Do Not Follow/Go Away	Simple (5)	Direct (5)	Random (6)	Pitch (7)	Stable (5)	Rectilinear (7)
Watch it/Look at it	Simple (5)	Direct (4)	Random (4)	Throttle (6)	Variable (3)	Rectilinear (4)
		Indirect (4)	Cyclic (4)	Roll (4)		
Investigate	Compound (5)	Indirect (5)	Random (5)	Roll (6)	Stable (4)	Combinational (4)
				Pitch (5)		
Caution	Compound (6)	Indirect (6)	Random (5)	Roll (4)	Stable (5)	Rectilinear (6)
Follow it/move towards	Simple (5)	Direct (6)	Random (7)	Pitch (6)	Stable (5)	Rectilinear (7)
Yes/Approval	Compound (6)	Indirect (6)	Cyclic (5)	Throttle (7)	Variable (6)	Rectilinear (6)
Landing	Simple (4)	Direct (4)	Random (7)	Throttle (7)	Decreasing (6)	Rectilinear (5)
	Compound (4)	Indirect (4)				
Delivery	Simple (4)	Direct(5)	Random (8)	Roll (4)	Stable (4)	Rectilinear (3)
	Compound (4)			Pitch (4)		
				Throttle (4)		

Three states here are considered similar to those from Firestone (landing, investigate, and watch it/look at it). “Landing” is referred to by the same name here. In both of these studies throttle and decreasing altitude are considered significant, with weaker support for direct.

The second is area of interest, which we map to “Investigate” here. For both of these we see roll and pitch as significant commands. Four of the motions here are also curvilinear, supporting the motion finding.

Finally, the third is attract attention, which we map to “Watch it/Look at it.” For this state, roll and throttle are the only characteristics that were considered significant for attract attention, and we see both of those represented here, with six out of eight motions containing throttle and four containing roll.

It should be noted the final two states do not perfectly map to states in (Firestone et al., 2019), but rather convey similar intents. In any case, the support seems reasonably strong for similarities in the structure of the designed motions indicating potential differences across states.

#### 8.4.3 Comparison to Phase 3

Once again, when comparing across these phases, the expectation for Phase 4 to show support for Phase 3 findings would be based on high-level similarities between the created and selected motions. Notably, the same state options from Phase 3 are presented here, with only “Do Not Follow/Do Not Pass/Restricted/Go Away” condensed down to “Do Not Follow/Go Away” differing.

This phase shows strong support for the idea that movement along the *z*-axis is distinguished as a characteristic of landing, with all participants having movement along the *z*-axis (six descend, two up-down). It also supports that “Follow it/Move Towards” should have large motions along the *y*-axis (six based on *y*-axis), although the motions are split (five and three) in terms of having an associated movement on the *z*-axis.

The recommendation to stay and watch a UAV was to minimize the amount of motion or have large altitude changes. Three of the states are presented as minimized motion, two yaw only and one circle defined as being only big enough to see movement. In addition to this, six participants include a throttle component, three of which were defined as moving a large amount (in these cases at least six feet). So we see some support for minimizing motion, but also overlap with the findings for landing. This is not unexpected and has been common across the studies where participants indicate an interest in watching a landing vehicle.

For “Yes” we again saw people associate up-down here, four provided basic up-down movements. In both phases, participants mentioned that this was because they associated the movement with nodding or yes in sign language.

In terms of the “Do Not Follow” state, we saw five of the participants design a motion that involved retreating (moving away) in some capacity, which is strange because many of the participants also designed a retreating motion to signify “Follow it/Move Towards.” Phase 3 found it likely that movement along the *x*-axis would mean to not follow, so this phase does not support that finding. It should be noted that Phase 4 was the first to include differences in the speed of motions, so given this as an option it appears that participants may use speed to differentiate the meanings.

Another finding from Phase 3 indicated that complex motions should also result in participants moving away from an area. These results are generally supported by the “Caution” state, which has six motions defined as compound.

Generally, we observe at least partial support for the findings in Phase 3 from the motions designed by participants in Phase 4. One notable exception is in “Do Not Follow/Move Away,” but this could also be due to the simplification of this state to exclude the idea of a restricted area after the conclusion of Phase 3.

## 9 Characteristics

During the free response analysis in Phase 2, we quickly noticed people were responding with feelings within the responses regardless of whether we asked for it. Considering the findings from (Cauchard et al., 2016), we hoped to elicit similar personality traits, but were curious how participants would respond to flight paths when not varying the speed and height characteristics as in that work. Explicitly including this question also allowed us to investigate if different flight paths would elicit similar or different personalities.

We presented 2 independent raters, who were not participants, with the data from Phase 1 and asked them to attempt to categorize the responses into the emotional states from (Cauchard et al., 2016): Dopey/Sleepy/Sad, Grumpy/Shy, Happy/Brave, and Scared/Stealthy/Sneaky. They had high agreement (Kappa =0.63 and above) but indicated difficulty with the task. Feedback from raters indicated that they felt they were making a lot of assumptions by categorizing into these states, since it was typically inferred from an unrelated response. In future phases, we explicitly asked the questions to the participants and with the goal to gain complementary information regarding the states being selected. In regards to the raters, the overwhelmingly popular (by more than three times) category for both of them when sorting Phase 1 responses was Happy/Brave.

### 9.1 Personality Scale Definition

**FIGURE 3:**
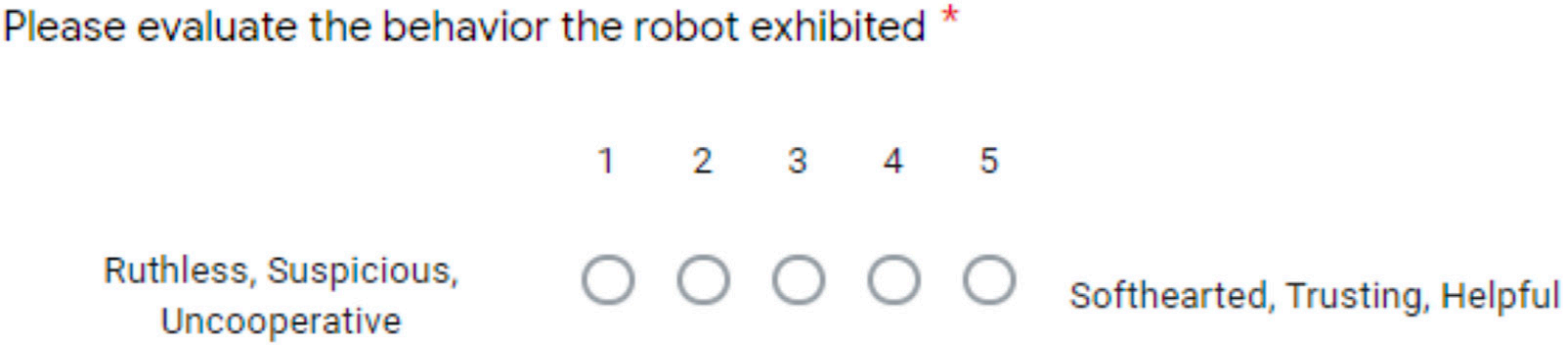
Example of personality question as displayed to the participants in Phase 1 and 3.

Modeled after (Spadafora et al., 2016; Cauchard et al., 2016) who presented the stereotypes of personality, each of the participants were given five scales they had to rank each of the videos using a 5 point Likert scale, pairing one extremity to the left side and the other to the right. The questions represented the “Big five” traits conveyed by two opposite poles: Openness to Experience, Conscientiousness, Agreeableness, Extraversion, and Neuroticism. Figure 3 is a visual example of how this was presented to participants, and Table 9 is the full list of presented characteristics.

**TABLE 9:** Big five opposing characteristics presented as anchors to the Likert scale.

1	5
Practical, conforming, interested in routine	Imaginative, independent, interested in variety
Disorganized, careless, impulsive	Organized, careful, disciplined
Ruthless, suspicious, uncooperative	Softhearted, trusting, helpful
Retiring, sober, reserved	Sociable, fun-loving, affectionate
Anxious, insecure, self-pitying	Calm, secure, self-satisfied

### 9.2 Phase 3: Personality Characteristics

Most commonly participants classified the videos with Practical/Conforming, Organized/Disciplined, and Calm/Secure characteristics. According to (Cauchard et al., 2016) this meant that almost all of them would classify as brave, which Cauchard further goes on to classify as an *Adventurer Hero* Drone type, regardless of the motion depicted.

X-shape and undulate stand out as being more imaginative, disorganized, ruthless, and anxious in nature than the other motions. These four characteristics don’t perfectly match any of the models, but come closest to Sad, Dopey/Sleepy, and Scared, which closely resemble the *Exhausted* Drone. This is interesting because in (Cauchard et al., 2016) they involve significant altitude changes, and thus would be unlikely to be designed this way to convey such a state. The difference in perceived personality is also interesting given that both of these flight paths still elicited the most common forced choice responses of “Move Away” and DNF.

#### 9.2.1 Personality Differences in Free Response vs. Forced Choice

Plus and Left-Right show opposite personalities when the participants were presented with free response options rather than forced choice. The responses for both motions showed significantly more imaginative traits assigned in free response, as categorized by the raters, and more practical in forced choice, as chosen by the participants. Again, this may be at least partially attributed to the experiment design as participants may be projecting their emotions onto what they see the UAV doing.

### 9.3 Phase 4: Personality Characteristics

During the online creation of participants’ motions, they were also asked to assign a UAV model to each state. Those responses are shown in Table 10.

**TABLE 10:** Applied characteristics.

	Sneaky spy	Adventurer hero	Anti-social	Exhausted	Other
Do not follow/Go away	0	1	6	0	1
Watch it/Look at it	2	3	0	0	1
Investigate	4	2	1	1	0
Caution	0	2	2	2	2
Follow it/Move towards	2	4	1	1	0
Yes/Approval	1	3	0	1	3
Landing	1	1	1	5	0
Delivery	1	4	0	1	1

Overall there is strong consensus for *Adventurer Hero*, which is the model most applicable to the results of Phase 3. Other states that diverge have converged to applicable archetypes, such as *Anti-Social* for “Do Not Follow/Go Away,” *Sneaky Spy* for “Investigate,” *Adventurer Hero* for “Follow it/Move Towards” and “Delivery,” and *Exhausted* for “Landing.” This lends support to both lines of work and calls for future studies explicitly linking the design characteristics from the designed motions here and the motion characteristics defined in (Cauchard et al., 2016).

There were still differences in the design of the motions when these motion characteristics were requested. For example, Cauchard places *Anti-Social* at about chest height and at an average speed. From our findings, “Follow it/Move Towards” motion had a large number of participants placing it at chest height with an average speed, but it is classified as *Adventurer Hero* by these participants. While none of the states have both a categorization of above head height and fast speed in this work, the closest resembling this is for “Yes/Approval,” which participants also classify as *Adventurer Hero* and which matches the recommendations of Cauchard. The final set of parameters in Cauchard are for *Exhausted* personality profile. For this, the speed is slow and the altitude is best understood to be waist or below in this case. This best matches Delivery, which is also classified as *Adventurer Hero* by these participants.

### 9.4 Phase 4: In-Person Characteristics

The participants that came in-person to complete their study were presented with the same labels presented in Table 9, but on a scale of 1-6 instead. All eight states had a classification of practical, organized, softhearted, and calm when sorted as (1,2,3) and (4,5,6). Practical/conforming, organized/disciplined, and calm/secure were the same characteristics applied to the majority of videos in Phase 3. In addition to these four classifications, the only state that had a significant result on the Retiring/Sociable scale was “Retiring, Sober, Reserved” for “Landing,” which had all five people classify it as a 3 (which is slightly agree on this scale). As before, this collection of characteristics doesn’t map perfectly to any of the models, but of the options 3 of the 4 map to brave, happy, and shy. Happy and brave are condensed into the *Adventurer Hero* Drone, and shy falls under *Anti-Social*, regardless of the requested state. This could again be due to the lack of co-design in the personality characteristics and the motion of the UAV, so this is suggested for explicit inclusion in future work.

## 10 Additional Exploratory Studies

Throughout these studies opportunities were presented to gain additional knowledge about both state labels and the effect of the different axes of motion within the flight paths. Some of these opportunities were investigated *via* small proto-studies that were run in-between the larger studies to better inform their design. These additional investigations were not central to the narrative above, but do provide complimentary information for completeness.

### 10.1 State Elicitation

Between Phases 2 and 3, an additional sixteen participants (not included in any of the above studies) were asked for 3-5 states they believe a UAV should convey. Eight of these participants were also asked what information they believed a UAV should be able to communicate to those not involved in the UAV’s operations. The question placement was counterbalanced between the beginning and the end of their study to see if participants provided more creative responses prior to applying given labels, or if they would provide the same states we provided if requested to provide states at the conclusion of the study. The placement of the request did not seem to have an effect overall. Regardless of placement, each of the participants submitted at least one of the states or labels that were included in the forced choice responses. The remaining portions of this study were not analyzed further due to poor responses. One lesson here, similar to that in the motion design and label creation categories is that creating prompts for participants which are open-ended enough to generate new ideas but narrow enough to provide overlap is a difficult endeavour.

### 10.2 Axis Investigation

After brief examination of the initial results of Phase 3 (the first 32/40 participants), we observed a seemingly consistent observation about the impact of the primary axis of motion. The initial observation was that motions moving mostly along the *x*-axis appeared as though they would elicit a blocked response, as demonstrated by all actions with the DNF choice were either significantly or solely on the *x*-axis. Whereas motions mostly on the *y*-axis seemed to rather encourage motion in that direction (to follow it), shown by front-back.

**FIGURE 4:**
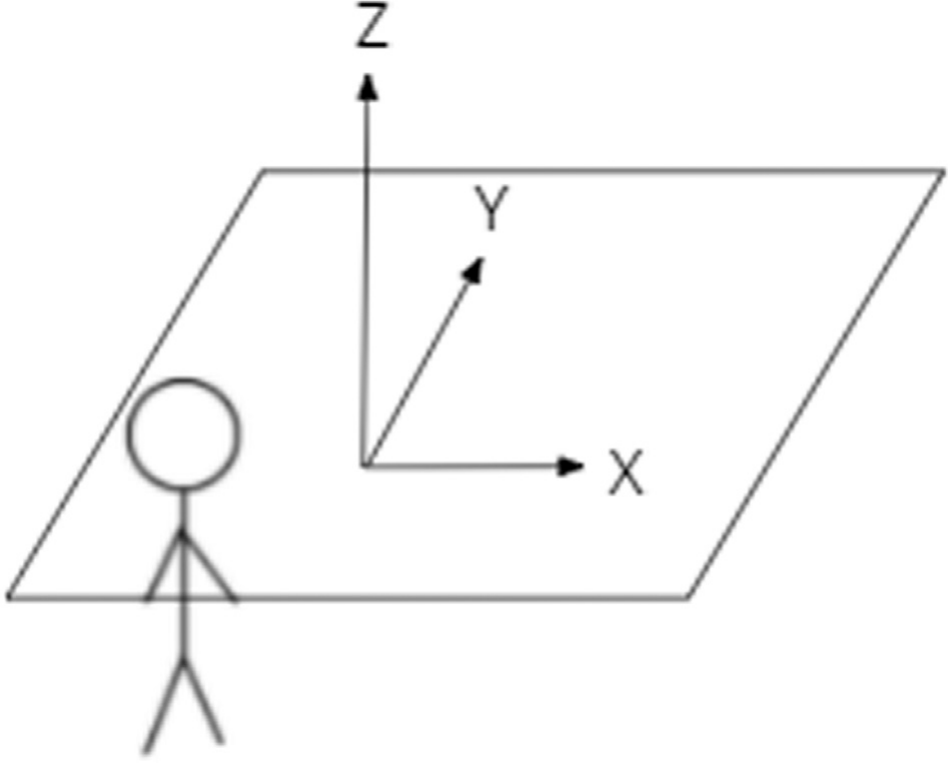
Direction of axes of motion relative to participant.

To test this observation, four of the motions that received the least amount of DNF categorizations from the first 32/40 participants in Phase 3 (front-back, straight descend, yaw, and diagonal descend) were replaced with four motions receiving the highest DNF classifications that had their primary axis of motion relocated from *x*-axis to *y*-axis (undulate, U-shape, X-shape, and horizontal figure 8). For these cases participants would then see both the original undulate on the x-z plane, in addition to an undulate on the y-z plane. This design was adopted to reduce any differences in this participant set. A visualization of the axes of motion relative to the participant is shown in Figure 4.

Ultimately there was not support for this initial observation within the exploratory dataset. The motions when rotated were still DNF, but we did observe a decrease in the intention to “Move Away” when compared to the earlier results. A different takeaway from these results is that it appears simplicity of the flight still holds a priority in effect, as with added complexity to the front-back motion we observed a change to a DNF state.

A noteworthy exception to the findings here is that horizontal figure 8, although initially classified DNF, when rotated received a tie for DNF and “Follow it” classifications. This could be due to the fact that this motion is unique from the others in that it moves a similar total x and y distance, with the distance on the *y*-axis from the participant being similar to that of the front-back motion. Another distinction this motion has from the other turned motions is a lack of motion on the *z*-axis. Overall this exploration is small and further study of these concepts would prove beneficial.

### 10.3 Phase 3 NARS Impact

The NARS questionnaire (Syrdal et al., 2009) contains questions asked on a Likert scale from 1 to 5, limiting participants’ scores within a given category to an average between 1 and 5. A score below 2 is considered positive, and a score above 3 is considered negative. Values between 2 and 3 are considered neutral.

After reviewing the states that were being presented to participants in Phase 3, they fell within three natural groupings. The first grouping contains states that can be associated with a more positive connotation, while also being states that could be considered as welcoming movement towards the UAV. The second grouping was neutral states, or states that may invite the viewer to be stationary. Finally, the third grouping was negative sentiment states, otherwise viewed as states that encouraged the viewer to move away from the UAV or discouraged interactions.

• Positive/Move Towards: “To Follow It/Move Towards,” “Yes,” “Welcome,” “Help,” “Follow it,” “Help it”
• Neutral/Stay: “Landing,” “Delivery,” “Watch it,” “Investigate”
• Negative/Move Away: DNF, “No,” “Caution,” “Move Away”

In total, participants provided 32 responses to questions that were prompted with this set of responses (16 responses for each question, 2 questions per video). Observing the correlation between people’s NARS scores and their chosen states, participants appeared more likely to choose a state from a given category based on whether they have a positive or negative NARS score. We observe that people with a NARS score classified as negative were more likely to pick negative states (mean:13.07, SD:4.7), and overall they were not as likely to choose one of the positive responses (mean:6.36, SD:3.4) t(26) = 4.27, *p* = 0.0002. Those with a positive NARS were likely to pick a positive state (mean:10, SD: 3.08) or negative state (mean:9.6, SD: 2.07) at about the same frequency t(8) = 0.24, *p* = 0.815. Both positive and negative NARS participants classified motions as one of the neutral options about 12 times on average [t (17) = 0.12, *p* = 0.907].

#### 10.3.1 Personality Traits and NARS

**FIGURE 5:**
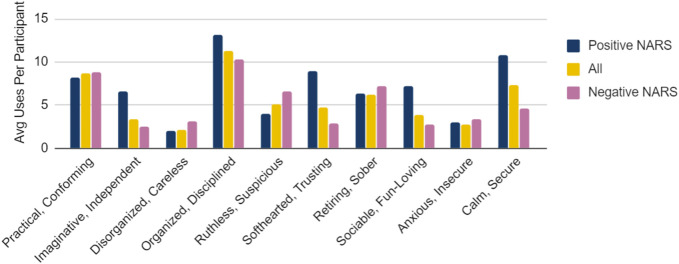
Average number of times a personality category was chosen by a participant based on their NARS score. The upper bound for number of uses is 16 per participant.

Another correlation was between the NARS scores and the personality traits assigned to the motions. The 14 participants who had a negative NARS score were more likely to define the UAV as conveying practical, disorganized, ruthless, retiring, and anxious characteristics as seen in Figure 5. Whereas the 5 participants who had a positive NARS score generally selected the opposite traits (imaginative, organized, softhearted, sociable, and calm). The average of all 56 participants fell within the neutral values on all of the traits.

We test the null hypothesis that there is not a relation between NARS scores and chosen personality traits using t-tests. Using a *t*-test for 2 independent means we see that 4 of these results show significance, meaning that there is a correlation between participants’ NARS score and categories chosen, with an *alpha* of 0.05. These four results are Imaginative t (17) = 2.35, *p* = 0.031, Softhearted t (17) = 3.51, *p* = 0.003, Sociable t (17) = 2.24, *p* = 0.038, and Calm t (17) = 3.13, *p* = 0.006.

There was no significant difference for practical t (17) = −0.24, *p* = 0.815, Disorganized t (17) = −0.53, *p* = 0.601, Organized t (17) = 1.28, *p* = 0.216, Ruthless t (17) = −1.38, *p* = 0.187, Retiring t (17) = −0.33, *p* = 0.749, and Anxious t (17) = −0.18, *p* = 0.862.

## 11 Discussion

This work investigated how the general public would perceive and respond to communicative flight paths from UAVs through an iterative refinement of both flight paths and state labels. The limitations, implications, recommendations, and our reflections on this work will be presented in this section.

### 11.1 Limitations

A limitation for all phases of this study is that the flight controller used did not maintain precision control of the altitude of the UAV over time, because of this the paths were slightly varied based on the battery levels at the time of a specific flight. This is primarily a concern for the videos since these motions were intended to be held at exactly the same center position. This was also less of a concern in-person as the in-person flights were typically much shorter than the 30 s, and if a significant change was noticed in the flight controller’s ability to hold the altitude the battery was just changed between demonstrations.

Although the biologically inspired motions chosen at the beginning of this research were expected to be culturally universal, the interpretations of the motions presented here are likely to be impacted by our participants’ culture. This is related to how cultures interpret body movements differently, as discussed in (Sogon and Masutani, 1989; Kita, 2009). This idea is particularly supported by Andonova and Taylor (2012) which discusses the cultural associations with specifically head nodding/shaking. Although head-nodding means approval or “yes” in many countries, it does not mean this universally. For example, Bulgaria has a reversed response pattern, where a vertical head movement means “no” and horizontal head movement means “yes.” Given the relatively limited representation of non-Americans in our studies this is important to note as it impacts the generalizability of these results to other cultures.

Another note is the differences in pay participants received throughout these phases. Pay for the tasks in Phase 0 was comparable to other similar tasks available on mTurk and to similar in-person studies at related universities at the time, but was ultimately determined to be too low. In addition to taking place in different years, this is why the pay was increased for future studies. It is possible that this may have impacted the quality of work provided by participants in Phase 0, as noted by (Litman et al., 2015).

Finally, the most significant limitation is that this work focuses exclusively on single-turn communication rather than multi-turn interactions such as in (Clinkenbeard, 2018; Csapo et al., 2012; Sidnell and Stivers, 2012). Future works should focus on more involved multi-turn interactions to better leverage the promise of this new communication modality.

#### 11.1.1 Video

A limitation of the work is that Phases 0 (Duncan et al., 2018), Phase 1, and Phase 3 were all limited to remote viewing using video recordings. While an effective preliminary method, the main concern is that it likely impacted participants’ ability to provide their true reaction, as there is almost always a difference between an expected reaction and a natural reaction. Another aspect of this work which might impacts participants’ ability to accurately predict their true reaction could be the lack of previous UAV interactions, particularly in a social context. This would naturally increase the gap between their expected reaction and actual reaction, or interpretation. This concern is further reinforced by the fact that every person who came in-person during Phase 4 had at least one motion they wanted to modify.

Video use also eliminates the ability to explore varied UAV size and sound effect. While participants were asked to always have their sound enabled for the videos, there was no sound verification. This likely means that some participants had their sound off, or at a barely audible level. This is a problem that needs to be further explored in-person because of the high level of impact these factors can have on presence, fear, and interest in the machine.

The height, size, and speed of the recorded motions presented were held relatively constant in these studies, as opposed to being varied to elicit emotional responses as in (Cauchard et al., 2016). This is a limitation because varying these factors may allow exploration of additional communicative functions (rushing, thoughtful, contemplative, etc). This was not an oversight, but a priority for the study to reduce those factors and see what emotions or states were elicited specifically from the flight paths. The impact of these factors is briefly explored in Phase 4, but warrants further investigation.

In general, when presented with a forced choice option, most people agreed that these states appropriately conveyed the message they were looking for or at least did not care to write-in a response. While we cannot know for sure which of these is true, however since the results of Phase 4 generally confirm those of Phase 3, the categories seem appropriate choices.

#### 11.1.2 Phase 4

A similar limitation (lack of choice and context) within the final phase was that the participants had to create the motions remotely over Zoom. As mentioned in further detail within Section 8, this reduced the fidelity of the participant interactions and raised questions from the participants about how and where this motion would be used. Some of this confusion could have been amplified because participants were purposefully not provided with any details about intended use or demonstrations so that they ideally create gestures that are able to be broadly applied. The danger in accidentally priming participants is discussed in more detail within that section, but also bears repeating here. Two excluded participants were each presented with curvilinear and rectilinear paths, respectively, as a demonstration before producing their own paths almost exclusively within those categories. Further investigation into appropriate context, demonstrations, and other priming mechanisms would be valuable when generating design characteristics for new technologies.

The size of the participant count within Phase 4 is also of note. There were limitations in having a larger participant pool participate in-person due to health and community regulations at the time of the study (in winter 2020). Thus the concept behind Phase 4 is support our larger online studies and provide possible paths for future work, rather than being a summative study to conclude the work.

### 11.2 Implications

We present an exploration into perceived communication, expected physical response, and emotional response to varied UAV flight paths. As a result of this, there are important practical implications discovered here for UAV developers and future researchers that may help to provide safe and knowledgeable interactions for the general public. This work indicates that people relatively easily associate motions applied in other situations onto UAVs, especially in the cases of Landing being conveyed with an altitude change, and a controlled up-down communicating “Yes.” If a UAV begins to move away from someone at a lower height and slower speed, it is highly likely to be understood to follow it, especially if the motion is dynamic (periodic yaw to “look back” at the person, or clearly going in a specific direction). Because we also saw Do Not Follow have a retreating motion, the context added by the speed and height of interaction become highly important.

We were able to elicit different personalities, as described by (Cauchard et al., 2016), without varying the underlying flight characteristics and thus extending that work. One of the more significant deviations from (Cauchard et al., 2016) is that the undulate motion is used as a prototype of *Adventurer Hero*, but the participants here classify that motion as one of few to be *Exhausted*. Overall, participants classified almost all motions as Brave, and in turn the UAV as an *Adventurer Hero* type, which held across both Phase 3 and 4 and in spite of the UAV base characteristics being more closely aligned with those of the *Anti-Social* Drone and *Exhausted* Drone.

Overall, the work presented here builds and presents aspects in each new phase that support previous findings with at least a low level of confirmation by leveraging early findings as a starting point for exploration in this iterative process.

### 11.3 Recommendations

A major recommendation which has been presented in recent sections and in the discussion so far has been in the need for study on how to situate requests to participants in designing interactions with novel technologies without priming their responses and while still producing convergent ideas. The work presented here was a first step towards identifying common expected communications and underlying assumptions about the meaning of different flight paths, but still leaves many questions open regarding height, speed, and place of interaction. A challenge throughout this work has been establishing underlying mental models of UAV flight paths without priming those models towards specific path components (as discussed in Section 8.1.4).

Another recommendation is to explicitly bridge the work between (Cauchard et al., 2016; Bevins and Duncan, 2021) in order to apply the personality models to the designed flight paths and understand any changes in participant perception. Given how underexplored the area of human-UAV interaction has been, this work has converged in an interesting and exciting ways to build upon these lines of inquiry.

In a more fundamental sense, we have recommendations on flight paths, which include the complexity of motion, leveraging other motions within a culture, and the need to include the speed/height characteristics in future studies. From our results we found that complex motions frequently indicated an intention to move away from the UAV and/or area whereas simplifying or minimizing the motion would encourage them to stay and watch the UAV. Participants also associate motions applied in other situations well onto UAVs, especially in the cases of “Landing” being conveyed with an altitude change, and a controlled up-down communicating “Yes/Approval.” If a UAV begins to move forward at a lower height and slower speed, it is highly likely to be understood to follow it, especially if the motion is dynamic (periodic yaw to “look back” at the person, or clearly going in a specific direction). Finally, as mentioned above, we note the need to have speed and height control to motivate a given context of interaction.

### 11.4 Reflection

An interesting result from the final Phase was the large amount of movement along the *y*-axis for the “Do Not Follow/Go Away” motions. At least one participant mentioned that if they were not supposed to follow the UAV then they would prefer that it depart the area (or at least their view). Contrast this to participants from Phase 3 where they perceived the “Go Away,” as more similar to a guarding or protecting motion seen in a variety of communication scenarios (such as basketball guarding, a patrol team or dog). The dissonance between the two could be from a change in the state description where “Do Not Pass” and “Restricted” were removed as a simplification between Phase 3 and 4. While the authors assumed this change would have little to no effect on the responses, if this were a correct assumption, it could be assumed that a movement in-front of a person would give off a message that an area is blocked/to not approach, and to communicate not to follow is more associated with a speed and height than a particular motion (i.e., too fast and high). These types of findings can lead to a perceived brittleness in the studies conducted and the generalizability of the findings, however they could also be a testament to the difficulty in defining interactions with a new technology. Many of the findings were consistent across a 3-years, four phase study that was meant to both build and challenge its earlier results.

Throughout this work the most popular and recognizable characteristics of motion seem to frequently mirror an already recognized motion in a variety of domains. We see this represented most prominently with yes being associated with up-down and landing being associated with a straight descend, in addition to the note about guarding above. Everyday people, regardless of their design ability, have seemingly pulled these characteristics from interactions across human, object, or animal movement, and applied it as being effective in human-UAV communication. This is promising for future studies in this area, particularly in the open areas identified to provide support among disparate lines of research within this new field.

While this work has limitations, it extends the state-of-the-art in understanding how people interpret aerial vehicle motions to assist in informing how they may most effectively communicate messages (both via targeted motions and messages people are expecting to receive) to people using the most fundamental communication method in their flight paths. Future work is necessary to build upon the results shown here, but this work has taken a meaningful step towards bringing together previous work and understanding what people perceive about these systems.

### 11.5 Future Work

The most well-motivated future work described here is to merge the lines of research discussed in Phase 4 with those presented in (Cauchard et al., 2016) via a larger study either online or in-person. This would provide a richer set of flight paths with clear guidance on speed, height, and personality expectations to communicate specific states. This work could also be extended to understand how those flight path characteristics are impacted by the location of interactions and context inherent in the location changes (expectations for indoor versus outdoor, home versus public spaces, etc.).

As motivated in the limitations section, this work is focused exclusively on either one way communications or, at best, single-turn interactions. Future work would benefit from understanding how to leverage these into multi-party or at least multi-turn interactions. Recommended additional studies described below will also contribute to this improved understanding of a more involved or robust interaction.

Some specific limitations of the current work that could be addressed through additional studies include: adding context, adapting from designers in other areas, understanding the perception changes from in-person to online interactions, and the impact of additional communication modalities. It would be interesting to explore how flight paths vary when participants are given a specific scenario or use case to see how they adapt for each situation. As in other design work, it may prove beneficial to explore having animators, or dancers create the motions, as they are already trained in thinking about how to have people interpret motion that communicates messages. An extension of specifically Phase 3 would be to run the motions from Phase 3 in-person to see the full effects of being near the UAV as opposed to just viewing it online. Other factors to explore in the future that would compliment this work include adding light components, as mentioned by participants throughout, or changing the vehicle design.

Briefly addressed above and in Phase 4 would be further separation of categories combined here for simplicity, specifically splitting the“Do Not Follow/Do Not Pass/Restricted/Go Away” category. While this category did provide general motivation, which was its purpose, it also appeared to be a catchall and may be better understood with separation of it into individual components. This was partially attempted by removing the restricted/go away between Phase 3 and Phase 4, it also appeared to lead to a large change in meaning and should have been split rather than simplified. Finally, a common note from in-person participants in Phase 4 was that they had imagined the motion would be more noticeable. This gap reinforces a rather simplistic understanding of UAV motion that we have (unsuccessfully) attempted to address in various phases. It is imperative to understand how to create a model of UAV flight, including the range of motion and inherent noise in the motion while not biasing the motions created by the participants. Perhaps this limitation could be addressed by providing a comprehensive explanation of typical UAV movement during describing the task or training participants on UAV flight characteristics, but there are concerns with priming responses when providing further details or demonstrations. This is a fundamental issue which needs to be addressed in future work to truly understand how to best leverage flight paths for communications.

## 12 Conclusion

Through this work we have been able to understand how participants would respond, both physically and emotionally, as well as better understand their perception of the messages naturally being conveyed within vehicle flight paths.

This work suggests that NARS can be an indicator of how a person may expect to respond and perceive the general sentiment of the message being conveyed. This work also indicates that people associate motions applied in other situations well onto UAVs. Especially in the cases of “Landing” being conveyed with an altitude change, and a controlled up-down communicating “Yes/Approval.” If a UAV begins to move forward at a lower height and slower speed, it is highly likely to be understood to follow it, especially if the motion is dynamic (periodic yaw to “look back” at the person, or clearly going in a specific direction). Because we also saw “Do Not Follow/Go Away” have a retreating motion, it’s highly important to note the need for speed and height situational control for proper context. Finally, flights crossing (moving along the *x*-axis) an area are likely to cause participants to avoid that area.

Finally, this work provides a roadmap to iteratively investigate the underlying communicative potential of new technologies while also raising significant questions about how to best elicit convergent states to communicate and common understanding of motion primatives. The discussion provided should be of keen interest to researchers investigating novel communication and to researchers in human-UAV interactions to understand where future work may have the most impact on bridging disparate investigations into this novel field.

## Data Availability

The raw data supporting the conclusion of this article will be made available by the authors, without undue reservation.
